# Inflammatory myopathy with anti-signal recognition particle antibodies: case series of 100 patients

**DOI:** 10.1186/s13023-015-0277-y

**Published:** 2015-05-13

**Authors:** Shigeaki Suzuki, Atsuko Nishikawa, Masataka Kuwana, Hiroaki Nishimura, Yurika Watanabe, Jin Nakahara, Yukiko K. Hayashi, Norihiro Suzuki, Ichizo Nishino

**Affiliations:** Department of Neurology, Keio University School of Medicine, Tokyo, Japan; Department of Neuromuscular Research, National Institute of Neuroscience, and Department of Clinical Development, Translational Medical Center, National Center of Neurology and Psychiatry, Tokyo, Japan; Department of Allergy and Rheumatology, Nippon Medical School Graduate School of Medicine, Tokyo, Japan; Department of Neurophysiology, Tokyo Medical University, Tokyo, Japan

**Keywords:** Signal recognition particle, Autoantibodies, Necrotizing myopathy, RNA immunoprecipitation, ELISA, Outcome

## Abstract

**Background:**

Anti-signal recognition particle (SRP) antibodies are used as serological markers of necrotizing myopathy, which is characterized by many necrotic and regenerative muscle fibers without or with minimal inflammatory cell infiltration. The clinical spectrum associated with anti-SRP antibodies seems to be broad.

**Objective:**

To describe the clinical characteristics, autoantibodies status, and neurological outcome associated with anti-SRP antibody.

**Methods:**

We studied clinical and laboratory findings of 100 patients with inflammatory myopathy and anti-SRP antibodies. Anti-SRP antibodies in serum were detected by the presence of 7S RNA using RNA immunoprecipitation. In addition, enzyme-linked immunosorbent assays (ELISAs) using a 54-kD protein of SRP (SRP54) and 3-hydroxyl-3-methylglutatyl-coenzyme A reductase (HMGCR) were also conducted.

**Results:**

The mean onset age of the 61 female and 39 male patients was 51 years (range 4–82 years); duration ≥ 12 months before diagnosis was seen in 23 cases. All patients presented limbs weakness; 63 had severe weakness, 70 neck weakness, 41 dysphagia, and 66 muscle atrophy. Extramuscular symptoms and associated disorders were infrequent. Creatine kinase levels were mostly more than 1000 IU/L. Histological diagnosis showed 84 patients had necrotizing myopathy, and apparent cell infiltration was observed in 16 patients. Anti-SRP54 antibodies were undetectable in 18 serum samples with autoantibodies to 7S RNA. Anti-HMGCR antibodies were positive in 3 patients without the statin treatment, however, were negative in 5 patients with statin-exposure at disease onset. All but 3 patients were treated by corticosteroids and 62 (77 %) of these 81 patients required additional immunotherapy. After 2-years treatment, 22 (27 %) of these 81 patients had poor neurological outcomes with modified Rankin scale scores of 3–5. Multivariate analysis revealed that pediatric disease onset was associated with the poor outcomes.

**Conclusion:**

Anti-SRP antibodies are associated with different clinical courses and histological presentations.

**Electronic supplementary material:**

The online version of this article (doi:10.1186/s13023-015-0277-y) contains supplementary material, which is available to authorized users.

## Background

Signal recognition particle (SRP), which is a ubiquitous cytoplasmic RNA protein consisting of 7S RNA and 6 proteins with molecular weights of 9, 14, 19, 54, 68 and 72 kD, mediates the translocation of newly synthesized protein across the endoplasmic reticulum. Anti-SRP antibodies were first discovered in the serum of patients with clinical polymyositis by the presence of 7S RNA detected by RNA immunoprecipitation [1–3]. RNA immunoprecipitation is a powerful method for the detection of various autoantibodies, including those against aminoacyl transfer RNA synthetase (ARS). There is another method for detecting anti-SRP antibodies: an immunoassay using a 54-kD subunit protein of SRP (SRP54) as the antigen [4]. Immunoassays using SRP54 such as enzyme-linked immunosorbent assays (ELISAs) are easily performed and have the advantage of allowing the screening of many serum samples. However, comparisons of the RNA immunoprecipitation method and the SRP54 immunoassay method have not been conducted.

Based on an accumulation of clinical observations, it was reported that anti-SRP antibodies are associated with the severe and refractory myositis and that they can be regarded as myositis-specific antibodies [1]. Histological diagnoses have confirmed a tight association between anti-SRP antibodies and immune-mediated necrotizing myopathy [5–8]. Anti-SRP antibodies are now used as serological markers of necrotizing myopathy, which is characterized by many necrotic and regenerative muscle fibers without or with minimal inflammatory cell infiltration. Since there is a lack of information regarding the inflammatory processes in muscle histology, the detection of anti-SRP antibodies merely suggests an immune-mediated mechanism. The clinical spectrum associated with anti-SRP antibodies seems to be broad [9–12]. We hypothesized that anti-SRP antibodies could define a distinct subset of inflammatory myopathies, and the purpose of present study is to report the clinical characteristics, autoantibody status, and neurological outcome of 100 patients with inflammatory myopathy with anti-SRP antibody.

## Methods

### Patients

From 1997 to 2012, we followed 17 patients with inflammatory myopathy with anti-SRP antibody at Keio University Hospital. Between January 2008 and September 2012, we identified another 83 patients with anti-SRP antibodies who were referred from all over Japan to Keio University Hospital or the National Center of Neurology and Psychiatry. Anti-SRP antibodies were detected by RNA immunoprecipitation. The diagnosis of inflammatory myopathy was based on the histological diagnosis with clinical, electrophysiological, and radiological findings. Clinical information was retrospectively obtained by the authors or provided by referring physicians. This study was approved by the Institutional Review Boards at Keio University and the National Center of Neurology and Psychiatry.

### Histology

Necrotizing myopathy was diagnosed based on the observation of many necrotic fibers as the predominant abnormal histological feature without or with minimal inflammatory cell infiltration [7, 8]. Sporadic inclusion body myositis was diagnosed by the identification of rimmed vacuoles with non-necrotic fibers invaded by mononuclear cells or increased major histocompatibility complex (MHC) class I expression. Polymyositis was diagnosed based on endomysial inflammation cell infiltrate surrounding or invading non-necrotic muscle fibers accompanied by ubiquitous MHC class I expression. Dermatomyositis was diagnosed by the identification of the presence of perifascicular atrophy [13, 14]. Other inflammatory myopathies were regarded as non-specific myositis including perimysial or endomysial inflammatory cell infiltration.

### RNA immunoprecipitation

**Fig. 1:**
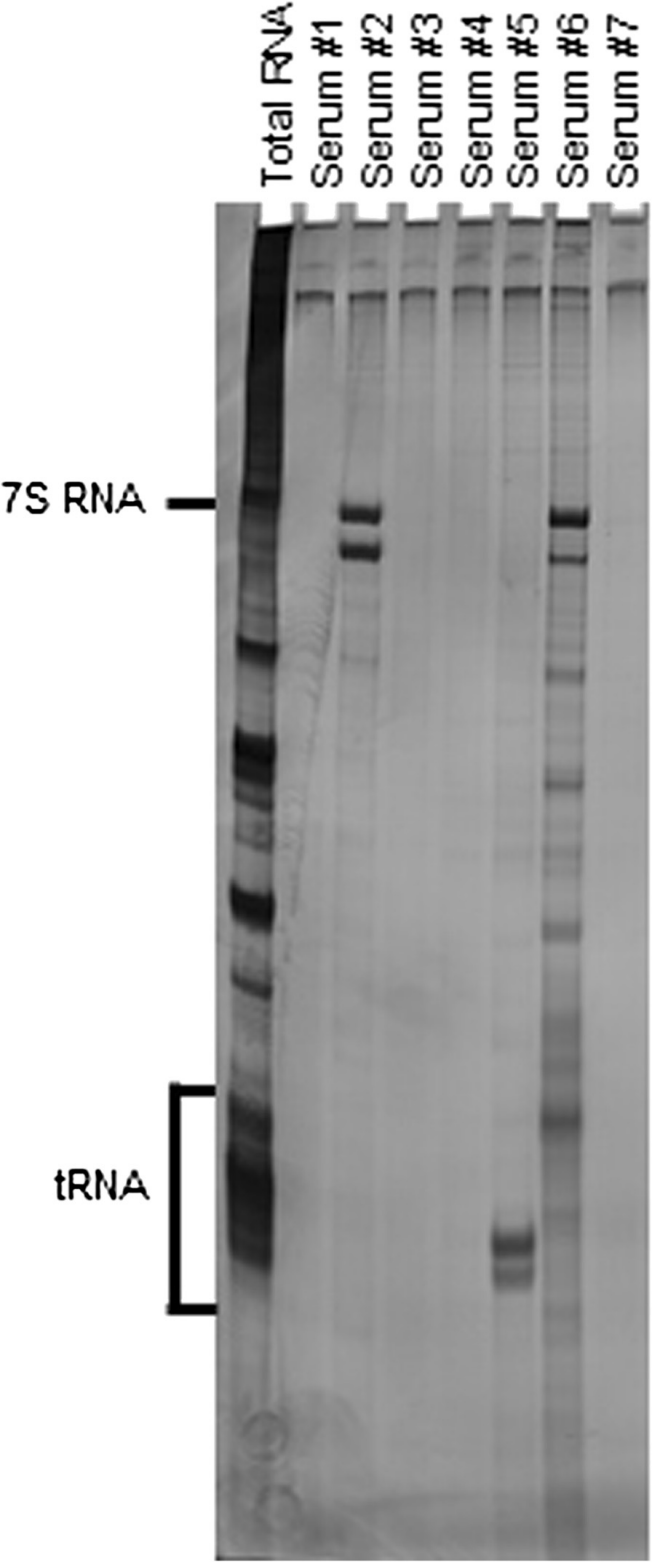
RNA immunoprecipitation assay. Urea and 8 % PAGE of phenol-extracted immunoprecipitation from HeLa cell extracts were developed with silver staining. Total RNA indicates 7S RNA and transfer RNA regions. The serum samples #2 and #6 contained anti-signal recognition particle (SRP) antibodies. In contrast, one of autoantibodies against aminoacyl transfer RNA synthetase, anti-PL-7 antibody (anti-threonyl-tRNA synthetase) was found in serum #5

Ten-μl of serum was mixed with 2 mg of protein A-Sepharose CL-4B (Pharmacia Biotech AB) in 500 μl of immunoprecipitation buffer and incubated for 2 h. After washing 3 times with immunoprecipitation buffer, antigen-bound Sepharose beads were mixed with 100 μl of HeLa cell extract (6 × 10 cell equivalents per sample) for 2 h, and then 30 μl of 3 M sodium acetate, 30 μl of 10 % sodium dodecyl sulfate, and 300 μl of phenol:chloroform:isoamyl alcohol (50:50:1, containing 0.1 % 8-hydroxyquinoline) were added to extract the bound RNA. After ethanol precipitation, the RNA was resolved by using a 7 M urea-8 % polyacrylamide gel, and the gel was silver-stained (Bio-Rad). Immunoprecipitated RNA located in the 7S RNA lesion was regarded as anti-SRP antibody (Fig. 1).

### ELISA

First, 96-well polyvinyl plates (Sumitomo Bakelite) were coated with recombinant SRP54 protein (Diarect) at 1 μg/ml diluted in phosphate buffered saline. The remaining blocking sites were blocked with 3 % bovine serum albumin. The wells were incubated with serum samples diluted 1:200 and subsequently with peroxidase-conjugated anti-human IgG (Jackson Immuno Research) diluted 1:100,000. The antibody binding was visualized by incubation with tetramethylbenzidine (1 mg/ml) in phosphate-citrate buffer. The reaction was stopped by 1 M sulfuric acid. The optical density at 450 nm (OD450) was read with an automatic plate reader (Biorad). Samples were tested in duplicate. In addition, autoantibodies to 3-hydroxyl-3-methylglutatyl-coenzyme A reductase (HMGCR) were measured using an ELISA based on the original method with some modifications [15].

We also used sera from 46 myositis patients without autoantibodies, 46 patients with myasthenia gravis, 40 patients with Duchenne muscular dystrophy, and 40 normal healthy volunteers as controls.

### Statistical analyses

Statistical analyses were performed using JMP version 9 statistical software (SAS Institute Inc.). Values of p < 0.05 were considered significant.

## Results

### Demographic features

**Fig. 2:**
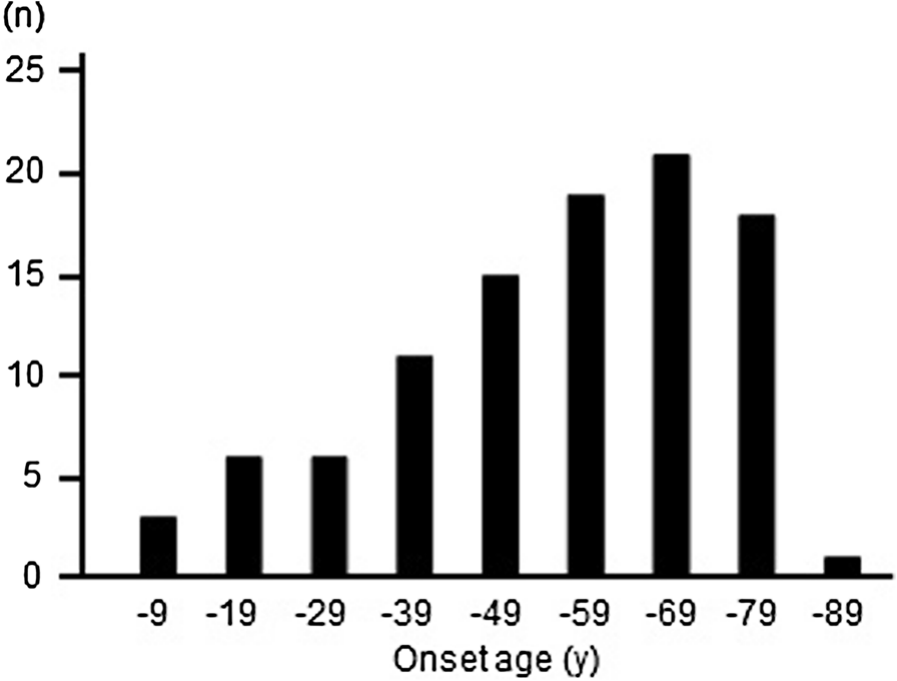
Distribution of onset ages in the 100 patients with inflammatory myopathy with anti- SRP antibody

The clinical features of the total 100 patients with anti-SRP antibodies are summarized in Table 1. There were 61 female, 39 males. The mean age of disease onset was 51.3 ± 19.3 years (range 4–82 years). The patients’ ages at disease onset were distributed widely, and peaked in the 60s (Fig. 2). Pediatric disease onset at age ≤ 16 years was found in 8 patients.

**Table 1:** Characteristics of the 100 patients with inflammatory myopathy with anti-SRP antibody

Findings	(n)
Females/males	61/39
Age at diseases onset	
Mean ± SD	51.3 ± 19.3
Age ≤ 15 years	8
Antecedent infection	7
Statin-exposure	5
Duration from the disease onset to the first examination	
≤12 months	77
>12 months	23
Initial symptoms	
Arms	19
Legs	67
Bulbar	7
Trunk	7
Muscle weakness	
Legs predominantly than arms	69
Severe limbs weakness	63
Laterality	16
Distal muscle dominant	3
Neck weakness	70
Dysphagia	41
Facial muscle involvement	10
Cardiac muscle involvement	2
Decreased capacity of respiratory function	12
Muscle atrophy	66
Scapular winging	10
Decreased deep tendon reflex	51
Myalgia	34
Extramuscular symptoms	
Fever	8
Skin rash	6
Arthritis	4
Raynaud phenomenon	7
Interstitial lung disease	13
Associated disorder	
Cancer	5
Rheumatic disease	9
Blood examination	
Creatine kinase (IU/L, mean ± SD)	6161 ± 4725
Elevated C-reactive protein	17
Antinuclear antibody positivity	5
Electromyography	
Spontaneous activity	41/86 (48 %)
Low-amplitude, short-duration motor unit potentials	79/86 (92 %)
Muscle images	
Atrophy on CT or MRI	46/79 (58 %)
Increased signals on T2 or STIR images	49/58 (84 %)
Histological diagnosis	
Necrotizing myopathy	84
Sporadic inclusion body myositis	0
Polymyositis	1
Dermatomyositis	1
Non-specific myositis	14
Autoantibodies	
7S RNA of SRP	100
54-kD protein of SRP	82
3-hydroxyl-3-methylglutatyl-coenzyme A reductase	3
Aminoacyl transfer RNA synthetase	0

SRP, signal recognition particle; STIR, short T1 inversion recovery

### Initial presentation

Antecedent infection was found in 7 patients. Statins were administered in 5 patients at disease onset. Inflammatory myopathy developed in 4 patients during the follow-up of another rheumatic disease. The durations of disease progression varied. The duration from the disease onset to the first examination was within 12 months in 77 patients, and ≥ 12 months in the other 23 patients.

With regard to the initial symptoms, weakness in the legs, such as difficulty in working or climbing stairs, was observed in 67 patients. Weakness in the arms was the initial symptom in 19 patients. The 7 patients with bulbar symptoms all suffered from difficulties in swallowing, but not in speaking. Among trunk muscles, neck weakness and muscle atrophy in the scapular muscles were reported in 5 and 2 patients, respectively.

### Clinical features

All patients had limb weakness. The distribution of weakness was proximal-dominant and symmetrical, and affected legs more than arms. Severe limbs weakness with the grade ≤ 3/5 assessed by manual muscle strength (Medical Research Council scale grade) was observed in 63 patients. Laterality of limb weakness was seen in 16 patients.

Seventy patients experienced neck weakness. Among them, dropped head was observed in 7 patients. Dysphagia was observed in 41 patients, and nasogastric tubes were necessary in 5 patients. Facial and cardiac muscle involvement was infrequent. The vital capacity percentage in the respiratory function test was decreased in 12 patients, 2 of whom needed mechanical ventilation. Neurological examinations revealed muscle atrophy in 66 patients: among them, scapular winging was seen in ten patients, which led physicians to make an initial diagnosis of facioscapulohumeral muscular dystrophy. Deep tendon reflexes were decreased or absent in 51 patients. Thirty-four patients reported Myalgia, especially in the early stage of disease.

With regard to extramuscular manifestations, the frequencies of fever, skin rash, arthritis, and Raynaud phenomenon were generally low. Chest CT revealed interstitial lung disease in 13 patients, all of whom had non-specific interstitial fibrosis, and their respiratory symptoms were generally mild. Malignancy (including lung, breast, stomach, ovarian and renal cancers) was discovered in 5 patients. Concomitant rheumatic diseases included Sjögren syndrome in 5 patients, systemic lupus erythematosus in 2, and rheumatic arthritis in 2 patients.

### Laboratory, electrophysiological, and radiological tests

With regard to the routine laboratory findings, serum creatine kinase levels were markedly elevated to more than 1000 IU/L in all but 2 patients (mean 6161 IU/L, range 735–21,544 IU/L). In addition, the median on peaked creatine kinase level was 4998 IU/L (25–75 % interquartile range 3354–7464 IU/L). Elevation in C-reactive protein (≥1 mg/dL) was seen in 17 patients. Positivity of antinuclear antibody (≥1:160) was detected in only 5 patients.

We assessed the findings of electromyography from 86 patients. Needle electromyography revealed positive sharp waves and/or fibrillation potentials in 41 (48 %) of the 86 patients. Patients with chronic progression tended to lack the spontaneous activity. In contrast, myopathic motor unit potentials such as short-duration and low-amplitude motor units with early recruitment were detected in 92 % of the 86 patients.

**Fig. 3:**
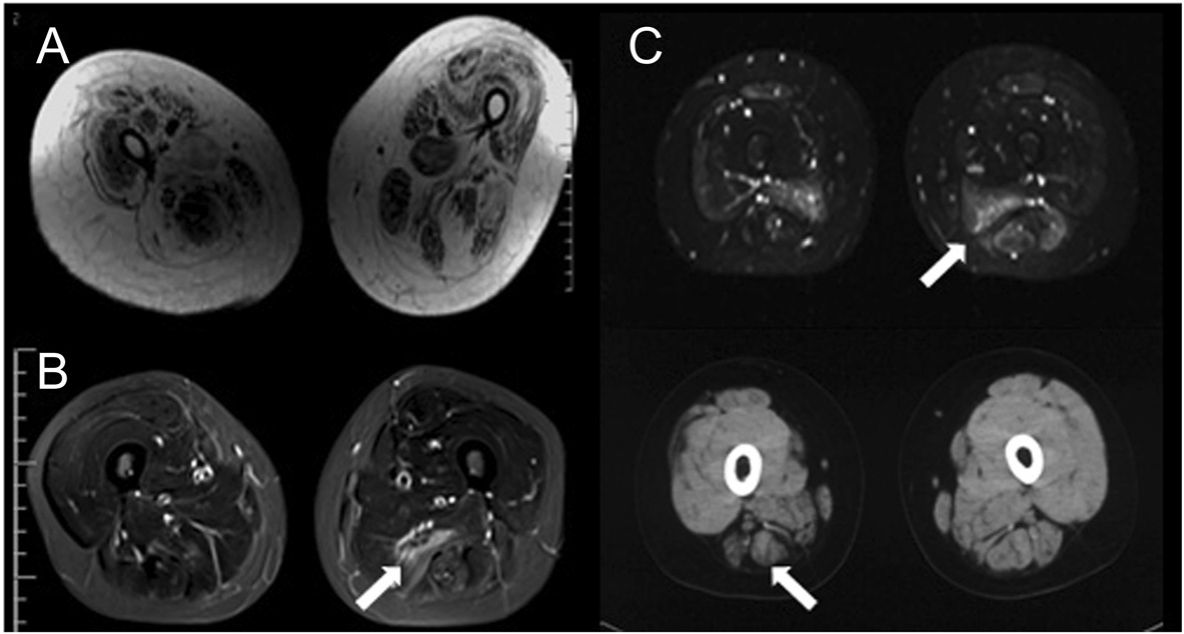
Muscle images of thighs in patients with anti-SRP antibodies. **(a)** Muscle atrophy on MRI T1 images. **(b)** High signal intensity on MRI STIR images. **(c)** Increased signals on MRI STIR images (upper) and muscle atrophy in CT (lower)

Information obtained by muscle CT or MRI was available for 38 and 58 patients, respectively. The distribution of inflammation and muscle atrophy was detectable in muscle CT or MRI in the thighs (Fig. 3). Muscle atrophy on CT or MRI was seen in 46 (58 %) of 79 patients. High-intensity signal in T2 or short TI inversion recovery (STIR) images were observed in 49 (84 %) of 58 patients.

### Histological diagnosis

With regard to the histological diagnoses, 84 patients had necrotizing myopathy, in which necrotic fibers > 1 % of total muscle fibers accompanied by regeneration fibers were observed. In contrast, there was apparent inflammatory cell infiltration in 16 patients. Inflammatory cell infiltration was regarded as positive when it was seen in the endomysial or perimysial regions, but not in the vicinity of necrotic fibers. Areas of invaded cells were in the endomysial region (*n* = 5), perimysial region (*n* = 7), or both (*n* = 4). The histological diagnoses of sporadic inclusion body myositis, polymyositis and dermatomyositis were made in 0, 1, and 1 patient, respectively. The other 14 patients had non-specific myositis.

### Detection of autoantibodies

All 100 serum samples were positive for anti-SRP antibodies using RNA immunoprecipitation. RNA immunoprecipitation revealed additional autoantibodies including anti-Ro/SS-A or anti-La/SS-B in 11, anti-U1RNP in 4, anti-Ku in 1, anti-Th/To in 1, and anti-ribosome in 1 serum sample. It was noted that no serum contained anti-ARS antibodies.

**Fig. 4:**
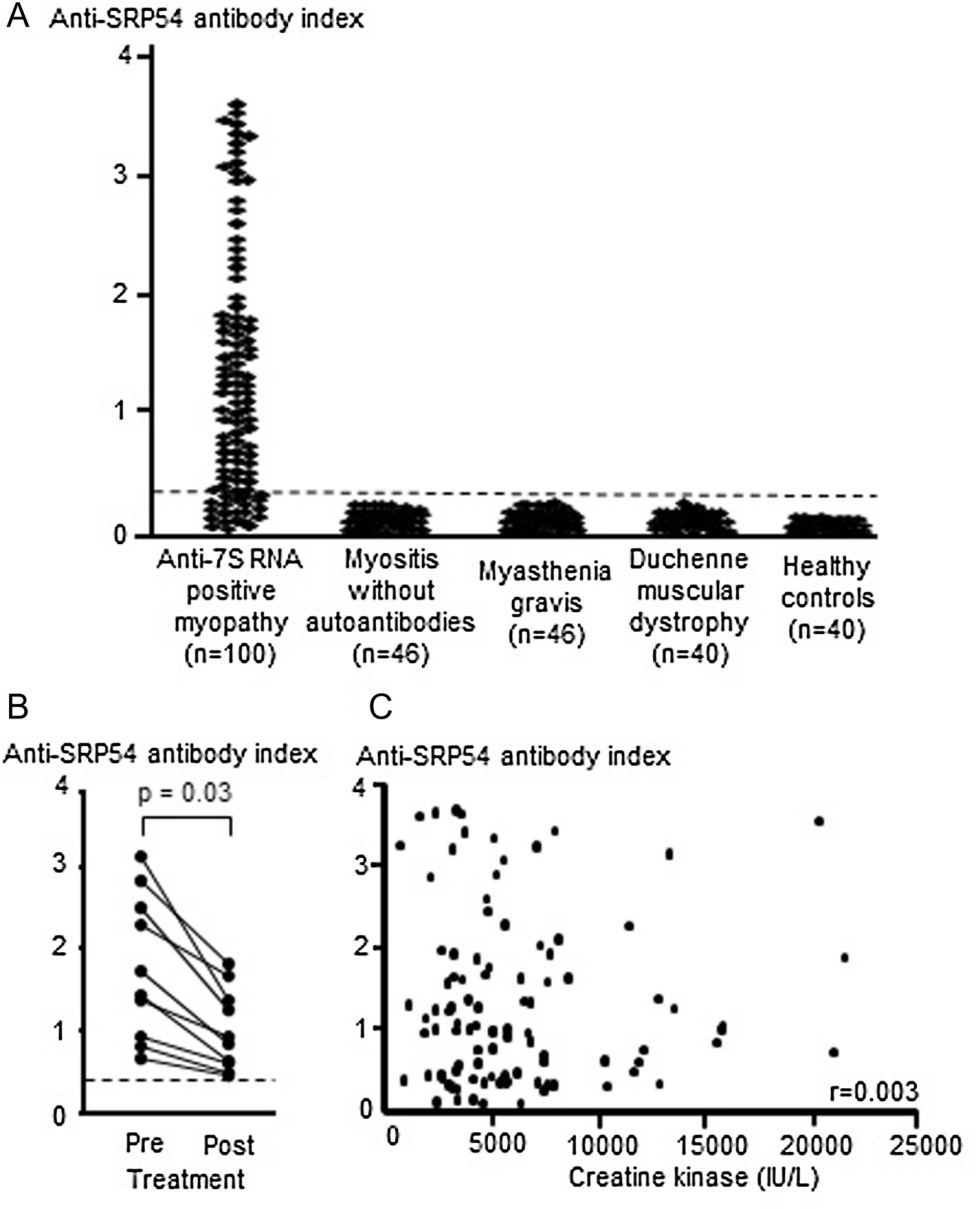
SRP54 enzyme-linked immunosorbent assay (ELISA). **(a)** Antibodies reactive with recombinant SRP54 protein by ELISA in sera from patients with anti-7S RNA positive myopathy, myositis without autoantibodies, myasthenia gravis, muscular dystrophy, and healthy controls. The cut-off level for positivity of anti-SRP54 antibody index is indicated by the broken line. **(b)** Serial changes of anti-SRP54 antibody index between pre- and post-treatment in 10 patients with anti-SRP antibodies. **(c)** Correlation of anti-SRP54 antibody index determined by ELISA and serum creatine kinase in 100 patients with anti-SRP antibodies

In addition to the gold standard detection method, anti-SRP antibodies were also evaluated by an ELISA using recombinant SRP54 protein as an antigen. The anti-SRP54 antibody index was calculated as the OD450 of the samples divided by the OD450 of the referential serum. A total of 272 serum samples were examined (Fig. 4). When the cut-off value was set as the mean + 5 × the SD of the healthy control sera (anti-SRP54 antibody index: 0.37), positivity for the anti-SRP54 antibody was detected in 82 serum samples with anti-SRP antibodies detected by RNA immunoprecipitation. The disease control samples (including those with myositis, myasthenia gravis and Duchenne muscular dystrophy) were all negative for anti-SRP54 antibody. Importantly, 18 serum samples had autoantibody against 7S RNA of SRP, but not against SRP54 protein.

We evaluated the serial changes in anti-SRP54 antibody index in ten serum samples. The anti-SRP54 antibody index decreased from pre-treatment to post-treatment (Fig. 4b). Since the longitudinal follow-up was not enough, we could not determine the association between the serial change of anti-SRP54 antibody index and creatine kinase or muscle weakness. In contrast, we evaluated the peaked serum creatine kinase values related with anti-SRP54 antibody index using same blood sample. There was no correlation between creatine kinase and anti-SRP54 antibody index in the 100 serum samples (r = 0.003, Fig. 4c).

To exclude the possibility of statin-induced myopathy, we also measured anti-HMGCR antibodies in the 100 serum samples. Anti-HMGCR antibodies were negative in the 5 patients with statin-exposure. Although anti-HMGCR antibodies were detected in 3 patients without the statin treatment, the titers of anti-HMGCR antibodies were much lower than those of the patients with statin-induced myopathy [15].

### Treatment

We further evaluated the clinical course of the 84 patients followed over 2 years. All but 3 of the 84 patients were treated with oral prednisolone. Two patients were proven to be positive for anti-SRP antibodies ten year after the disease onset. They suffered from the progression of muscle weakness during the first 3–5 years, and then the disease activity stopped. The remaining patient developed a cerebral infarction soon after undergoing the muscle biopsy.

In addition to prednisolone, 62 (77 %) of 81 patients required additional immunotherapy, including intravenous immunoglobulin (*n* = 33), intravenous methyl-prednisolone plus therapy (*n* = 32), tacrolimus (*n* = 22), methotrexate (*n* = 11), azathioprine (*n* = 11), cyclosporine (*n* = 9), intravenous cyclophosphamide (*n* = 3), or plasma exchange (*n* = 3).

### Clinical course

There were 3 types of clinical courses. In the first type, 26 (32 %) of 81patients responded well to immunotherapy without neurological deficits, although they required 2–3 months to respond to treatment. In the second type, 45 patients (56 %) were refractory to various immunotherapy regimens. The immunotherapy resulted in a decrease of the patients’ creatine kinase levels, but the recovery of muscle weakness was incomplete. These patients required long-term immunotherapy and suffered from side effects. Finally, the remaining ten (12 %) patients showed progressive muscle weakness. Their response to immunotherapy was minimal.

### Neurological outcome

The patients’ neurological outcomes were assessed at 2 years after the initiation of immunotherapy using modified Rankin Scale. Of the 81 patients treated with immunotherapy, 22 (27 %) had difficulties in their daily living graded as modified Rankin Scale scores 3–5. We divided these 81 patients into 2 groups: those with a good outcome, defined as a modified Rankin Scale score of 0–2 (*n* = 59) and those with a poor outcome, defined as a modified Rankin Scale of 3–5 (*n* = 22). We compared the clinical features before immunotherapy between the 2 groups (Table 2).

**Table 2:** Comparison of clinical features of 81 patients with good or poor outcomes

	Good outcome	Poor outcome	p
	(*n* = 59)	(*n* = 22)	
Females	35 (59 %)	16 (73 %)	0.3
Age at disease onset ≤ 15 years	1 (2 %)	6 (27 %)	0.0003
Disease progression > 12 months	12 (20 %)	8 (36 %)	0.1
Clinical characteristics			
Legs predominantly than arms	43 (73 %)	16 (73 %)	1.0
Severe limbs weakness	31 (53 %)	20 (91 %)	0.001
Neck weakness	37 (63 %)	17 (77 %)	0.2
Dysphagia	20 (34 %)	14 (63 %)	0.02
Muscle atrophy	32 (54 %)	20 (91 %)	0.002
Decreased deep tendon reflex	28 (47 %)	15 (68 %)	0.1
Myalgia	19 (32 %)	7 (32 %)	1.0
Interstitial lung disease	12 (20 %)	0 (0 %)	0.02
Laboratory findings			
Creatine kinase (IU/L)	6181 ± 4313	7079 ± 5263	0.5
Elevated C-reactive protein	7 (12 %)	8 (36 %)	0.01
Anti-SRP54 antibody index	1.4 ± 1.0	1.3 ± 1.1	0.8
Lymphocyte infiltration in histology	11 (19 %)	3 (14 %)	0.6

We found that pediatric disease onset, severe limb weakness, dysphagia, muscle atrophy, absence of interstitial lung disease, and elevated C-reactive protein were associated with the poor outcome. Moreover, multivariate logistic analyses revealed that pediatric disease onset was the only independent factor associated with the poor outcome (*p* = 0.003, odds ratio: 28.4, 95 % confidential interval 2.82–845).

Based on the result of multivariate logistic analyses, the onset age was an important factor for anti-SRP myopathy. We compared clinical and laboratory characteristics between 13 patients with younger onset and 68 patients with older onset using the cutoff age of 30 years (Additional file 1). The younger onset was associated with chronic disease progression, severe limbs and neck weakness and muscle atrophy. The neurological outcome was more severe in the younger onset compared with the older onset.

### Cause of death

Among 17 patients followed at Keio University Hospital for a longer follow-up, 7 patients died during the clinical course (age 74 ± 11 years). The side effects of the long-term use of corticosteroids may have been responsible for the death of 5 patients (cerebral infarction in 2 patients, ischemic heart diseases in 2, and bacterial pneumonia in 1 patient). The remaining 2 patients died of lung cancer and intestinal lung disease, respectively.

## Discussion

The present case series of 100 patients with inflammatory myopathy with anti-SRP antibody provided the following findings: (i) patients of all ages were affected; (ii) neurological symptoms were characterized by severe limb, trunk, and bulbar muscle weakness with atrophy; (iii) histological diagnoses showed 84 patients had necrotizing myopathy; (iv) anti-SRP54 antibodies were undetectable in 18 serum samples containing autoantibodies to 7S RNA of SRP; and (v) pediatric disease onset was associated with the poor neurological outcome. Taken together, these results led us to conclude that anti-SRP antibodies can be used determine a distinct subset in inflammatory myopathy (anti-SRP myopathy).

The diagnosis of anti-SRP myopathy is based on both the detection of anti-SRP antibodies in patients’ serum and the histological diagnosis of inflammatory myopathy, usually necrotizing myopathy. Ancillary tests and results including markedly elevated serum creatine kinase, electromyography, and muscle images support the diagnosis. Since the disease presentation and progression are variable, the clinical diagnosis is sometimes difficult. The chronic progression of anti-SRP myopathy accompanied by younger onset, severe muscle weakness and atrophy mimics muscular dystrophy. When a patient’s muscle weakness appears to be progressing faster than expected, as in muscular dystrophy, it may be worth testing for anti-SRP antibodies. The broad clinical presentation of SRP autoimmunity requires an expanded consideration of the anti-SRP antibody detection test in patients of all ages.

Necrotizing myopathy is a heterogeneous pathological category including autoantibody-mediated, drug-induced, paraneoplastic and viral infections [7, 8]. The presence of anti-SRP antibody is the most frequent etiology. We recently reported that 34 (53 %) of 64 patients with necrotizing myopathy had anti-SRP antibodies [16]. Anti-HMGCR and anti-ARS antibodies are also possible candidates as serological markers of necrotizing myopathy [8]. Anti-HMGCR antibodies were first known as markers of statin-induced myotoxocicity; however, they were also found in patients without statin-exposure [15, 17]. In the present study, although 5 patients received statins at the disease onset, they all had anti-SRP antibodies but not anti-HMGCR antibodies. Moreover, anti-ARS antibodies were not detected in our 100 patients with anti-SRP antibodies. The strength of present study is that we screened these 3 autoantibodies using RNA immunoprecipitation and ELISAs. We estimate that 3 types of autoantibodies may be independently attributed to the immune-mediated pathogenesis of necrotizing myopathy.

We detected anti-SRP antibodies by 2 different methods: RNA immunoprecipitation and an SRP54 ELISA. Benveniste et al. developed an addressable laser bead immunoassay using SRP54, and they demonstrated that the levels of anit-SRP54 antibodies were associated with the clinical course [12]. However, epitopes of anti-SRP antibodies are not always located in SRP54. Valiyil et al. demonstrated that 3 of 8 patients with anti-SRP antibodies did not have the autoantibodies to SRP54 [18]. In the present study, we have shown that 18 serum samples with autoantibodies to 7S RNA of SRP were negative for anti-SRP54 antibodies. It should be emphasized that false-negative results on an SRP54-specific immunoassay can be misleading. In contrast, RNA immunoprecipitation can recognize the conformational epitopes of the SRP complex, although the procedure is technically difficult and requires some technical skills and cultured cell extracts.

The neurological outcome of the patients with anti-SRP myopathy was unsatisfactory regardless of the combination of immunosuppressive agents. Our analyses revealed that pediatric disease onset was the most significant factor associated with poor neurological outcomes, suggesting that the dysfunction of the regeneration process of muscle fibers may be important in the pathogenesis of anti-SRP antibodies. In addition, we observed there was no correlation between the interstitial lung disease and poor neurological outcome. The severity of interstitial lung disease seemed to be mild. We thought decreased capacity of respiratory function was due to respiratory or trunk muscle weakness. In contrast, the elevated C-reactive protein was associated with poor neurological outcome. Increased levels of C-reactive protein were probably due to the systemic inflammation of anti-SRP myopathy. However, severe neurological deficits in SRP-positive patients are not related to survival.

Other autoantibodies have been found to be closely associated with the lethal condition, including anti-melanoma differentiation-associated gene 5 antibodies with rapidly progressive interstitial lung disease, anti-transcription intermediary factor 1 antibodies with malignancy, and anti-mitochondrial antibodies with cardiac involvement [19–21].

Our study has some limitations. First, the entry of patients in the present study was based on the referrals from other institutions. The frequencies of anti-SRP antibodies were found to be 5 %–8 % of the clinical diagnoses of myositis. In contrast, the frequency was up to 20 % of patients using the strict criteria of histological diagnosis [16]. We suspect that the actual prevalence of anti-SRP antibodies depends on whether the population is defined by clinical or histological criteria. Second, we cannot determine the best regimens of immunotherapy for anti-SRP myopathy. However, we emphasized that our treatment experience of 100 patients with anti-SRP myopathy suggested the valuable information. The combination of oral corticosteroids and intravenous immunoglobulin was effective as the first-line therapy. Additional immunosuppressive agents were also used for the purpose of reducing side effects of corticosteroids. The removal of anti-SRP antibodies by plasma exchange was considered as the second-line therapy.

## Conclusion

Anti-SRP antibodies are associated with different clinical courses and histological presentations. Further studies should clarify the pathogenesis of anti-SRP antibodies.

## Additional file

Additional file 1:
**Comparison of clinical features between younger and older onset.** Clinical and laboratory characteristic between 13 patients with younger onset and 68 patients with older onset using the cutoff age of 30 years were compared.
